# A study of satisfaction with research capacity development of Master of Public Health and its influencing factors

**DOI:** 10.3389/fpubh.2023.1263684

**Published:** 2023-09-20

**Authors:** Bin Hu, Haitao Zhou, Yue Wang, Li Zheng, Min Li

**Affiliations:** School of Public Health, Xuzhou Medical University, Xuzhou, Jiangsu, China

**Keywords:** Master of Public Health, scientific research capabilities development, satisfaction, fostering, research

## Abstract

**Objective:**

To understand the satisfaction and the current status of the training of scientific research ability of Master of Public Health students in universities, analyze the influencing factors and explore their solutions, in order to provide reference for improving the scientific research ability of Master of Public Health students.

**Methods:**

A questionnaire was used to survey Master of Public Health students in nine universities. The data were analyzed by descriptive statistics and multifactor logistic regression.

**Results:**

The overall satisfaction self-rating score for research capacity of Master of Public Health students was (3.29 ± 0.87), with the highest score for mentor exchange (3.78 ± 0.81) and the lowest score for subsidized treatment (1.86 ± 0.96). Satisfaction with the practice unit and school dimensions was significantly lower than the mentor dimension. The length of practice in the practice unit, subsidy treatment, importance of research capacity development, development tendency, and current status of research were influential factors contributing to the difference in satisfaction with research capacity development of Master of Public Health students (*P* < 0.05).

**Conclusion:**

The overall scientific training quality of Master of Public Health students is high, but there are still many aspects that need to be solved. Colleges and universities should join the funding system adapted to the practice process of Master of Public Health students and guarantee the construction of the system of student subsidy treatment. Secondly, they should strengthen the construction of public health supervisory team and improve the frequency and quality of scientific research exchanges between teachers and students. In addition, colleges and universities should improve the existing research incentives and policies, and adopt multiple forms and types of research incentives. Finally, colleges and universities should improve the research training system and the construction of research resources for Master of Public Health.

## 1. Introduction

In 2022, the General Office of the State Council issued a circular on the “14th Five-Year Plan” for national health, stating that it is necessary to build a strong public health protection network, improve the ability of disease prevention and control, and improve the monitoring and early warning mechanism. The guiding opinion of the General Office of the State Council on accelerating the innovation and development of medical education points out that it is necessary to accelerate the construction of a high-level public health personnel training system, and to strengthen the cultivation of practical scientific research ability of Master of Public Health professionals. Therefore, the reform of the teaching content and scientific research requirements of Master of Public Health has become the primary task of medical schools.

Master of Public Health refers to high-level talents who should have strong public health professional ability and professionalism, and apply to health service departments such as CDC, customs, community health service centers, hospitals and so on (1). However, the development of Master of Public Health students in China is relatively late, the faculty and teaching mode are still immature, and the training program of Master of Public Health students still needs to be effectively adjusted. Satisfaction with scientific research ability cultivation is an important comprehensive evaluation index for all aspects of scientific research ability cultivation in graduate school. Satisfaction affects the motivation of Master of Public Health students, and it also reveals the shortcomings of the current stage of medical education. This study investigates the satisfaction of the participants in various aspects of the process of developing the research capacity of Master of Public Health students and their influencing factors, and analyzes the current problems faced by Master of Public Health students in the development of their research capacity, with a view to providing a reference for improving the research capacity of Master of Public Health students.

## 2. Object and method

### 2.1. Study population

Stratified sampling method was used in this study. Thirty-six of the 55 colleges and universities in East China whose school type is medical university or contains the secondary discipline of public health were stratified according to the geographical distribution of North, Central, and South, and sampling was conducted in the ratio of 1/4, and finally nine medical higher education institutions were selected as the sample. A questionnaire survey was conducted in February-April 2023 using the form of online survey with the principle of voluntary completion for the Master of Public Health of grades 2020 to 2022, and 967 questionnaires were recovered, of which 925 were valid questionnaires, with a recovery validity rate of 95.66%.

### 2.2. Methodology of the study

In this study, a self-administered questionnaire was used to conduct a questionnaire survey for Master of Public Health students. The questionnaire included three aspects of basic information, research situation, and satisfaction with the development of research ability. Basic information includes gender, age, and academic year of study. The research section includes the time invested in research, the number of scholarly books read per month, the number of papers published, the participation in academic activities, and the participation in academic conferences. The satisfaction scale of scientific research capacity development is based on the reference of domestic and foreign related studies (2, 3), while modified with the characteristics of scientific research capacity development of Master of Public Health students and experts' opinions, and the scale is tested for reliability and validity. The Cronbach's alpha coefficient is 0.876 which is >0.6, and the KMO coefficient is 0.892 after rotating, the cumulative variance explanation rate is 72.58. The reliability and validity of the Master of Public Health Research Competency Development Satisfaction Scale were good. The Likert five-point scale was used, and the value “5-1” was assigned to very satisfied, basically satisfied, average, dissatisfied, and very satisfied, respectively.

### 2.3. Statistical methods

SPSS22.0 statistical software was used to process the data, and descriptive statistics, one-way ANOVA and multifactor logistic regression analysis were used. The difference was considered statistically significant at *P* < 0.05.

## 3. Results

### 3.1. Basic situation of Master of Public Health

The survey of 925 Master of Public Health, mainly including the classes of 2020 to 2022, including epidemiology and health statistics, labor and environmental hygiene, nutrition, MPH and other public health directions, the basic situation is shown in Table 1.

**Table 1:** Basic information on Master of Public Health (*n* = 925).

**Characterization**	**Classifications**	**Sample**	**Proportion (%)**
Gender	Male	320	34.59
	Female	605	65.41
School year	First postgraduate year	375	40.54
	Second postgraduate year	325	35.14
	Third postgraduate year	225	24.32
Age	20–24	780	84.32
	24–30	120	12.97
	30~	25	2.70
Whether the undergraduate degree is a medical degree	Yes	445	48.11
	No	480	51.89

### 3.2. Satisfaction of Master of Public Health students' research capacity development

The score of “1-2” was defined as “low satisfaction”, “3” as “basic satisfaction”, and “4-5” as “high satisfaction”. A score of “4-5” was defined as “high satisfaction”. The total mean score of satisfaction with the development of research capacity of Master of Public Health was (3.29 ± 0.87), which exceeded the medium intensity of “basic satisfaction”, but did not reach the higher satisfaction level. The least satisfaction was subsidized treatment (1.86 ± 0.96), followed by research incentive system (2.98 ± 0.83), except for that, all items reached basic satisfaction. The highest level of satisfaction is the effect of research guidance in mentor exchange, and the satisfaction level of each item in the part of faculty exchange is higher than 3.5, which is a high level of satisfaction. See Table 2.

**Table 2:** Satisfaction with the development of research capacity in Master of Public Health *n* (%).

**Entry**	**Very dissatisfied**	**Dissatisfied**	**Generally**	**Satisfied**	**Very satisfied**	**Score ($\bar{x}\;\pm$s)**
**Communication with graduate tutor**						
Time and energy	21 (2.27)	69 (7.46)	378 (40.86)	412 (44.54)	45 (4.86)	3.47 ± 0.89
Effectiveness of mentoring	14 (1.51)	49 (5.30)	312 (33.73)	457 (49.41)	93 (10.05)	3.78 ± 0.81
Research capacity	10 (1.08)	34 (3.68)	262 (28.32)	429 (46.38)	190 (20.54)	3.68 ± 0.84
Intra-group research activities	17 (1.84)	46 (5.97)	317 (34.27)	410 (44.32)	135 (14.59)	3.64 ± 0.87
**Practice units**						
Regulatory system	46 (4.97)	104 (11.24)	428 (46.27)	290 (31.35)	57 (6.16)	3.23 ± 0.92
Research resources	31 (3.35)	92 (9.95)	437 (47.24)	315 (34.05)	50 (5.41)	3.29 ± 0.85
Academic exchange	25 (2.70)	74 (8.00)	452 (48.86)	326 (35.24)	48 (5.19)	3.34 ± 0.81
Subsidized treatment	401 (43.35)	298 (31.22)	168 (18.16)	47 (5.08)	11 (1.19)	1.86 ± 0.96
**School education**						
Training management	47 (5.08)	98 (10.59)	458 (49.51)	285 (30.81)	37 (4.00)	3.18 ± 0.86
Research courses	37 (4.00)	112 (12.11)	436 (47.14)	310 (33.51)	30 (3.24)	3.22 ± 0.84
Academic lectures	22 (2.38)	26 (2.81)	494 (53.41)	262 (28.31)	121 (13.08)	3.49 ± 0.83
Research incentive system	79 (8.54)	168 (18.16)	415 (44.86)	212 (22.92)	51 (5.51)	2.98 ± 0.83

### 3.3. Current status of research situation of Master of Public Health

The results of the survey showed that of the 925 Master of Public Health students, 251 (27.14%) thought that research ability was very important, 464 (50.16%) thought that it was important, 156 (16.86%) thought that it was average, 24 (2.59%) thought that it was unimportant, and 30 (30) thought that it was very unimportant (3.24%). Regarding the survey on developmental tendencies, 235 people (25.41%) thought that practical skills should be the main focus, 270 people (29.19%) thought that scientific research skills should be the main focus, and 420 people (45.41%) thought that both scientific research and practical skills should be emphasized. Twenty-seven people (2.92%) surveyed the status of scientific research is very good, 89 people (9.62%) surveyed the status of scientific research is good, 627 people (67.78%) surveyed the status of scientific research is average, 117 people (12.65%) surveyed the status of scientific research is not good, and 65 people (7.03%) surveyed the status of scientific research is very bad. See Table 3.

**Table 3:** Satisfaction scores of the current status of the research situation of Masters in Public Health.

**Entry**	**Sample *n* (%)**	**Satisfaction rating**	***F***	***P***
**Importance of research skills**			13.416	< 0.001
Very important	251 (27.14)	3.43 ± 0.69		
Important	464 (50.16)	3.36 ± 0.55		
Not important	156 (16.86)	3.06 ± 0.55		
Not important	24 (2.59)	2.89 ± 0.57		
Very unimportant	30 (3.24)	3.29 ± 0.56		
**Research system**			10.67	0.047
Good	654 (70.70)	3.54 ± 0.49		
Average	241 (26.05)	3.24 ± 0.52		
Poor	30 (3.24)	3.01 ± 0.61		
**Incentive system**			14.65	< 0.001
Better	698 (75.46)	3.37 ± 0.43		
Poor	227 (24.54)	2.87 ± 0.56		
**Duration of practice in the unit**				
3 months and below	126 (13.62)	2.91 ± 0.64	11.371	< 0.001
3–6 months	235 (25.41)	3.15 ± 0.59		
6 months and above	564 (60.97)	3.42 ± 0.56		
**Subsidized treatment**				
1,000 and less	697 (75.35)	2.97 ± 0.53	14.324	< 0.001
1,000–2,000	189 (20.43)	3.01 ± 0.54		
2,000–3,000	21 (2.27)	3.21 ± 0.51		
3,000 and above	18 (1.95)	3.32 ± 0.55		
**Developmental orientation**			12.246	< 0.001
Practical skills	235 (25.41)	3.27 ± 0.58		
Research-oriented	270 (29.19)	3.06 ± 0.63		
Research and practice at the same time	420 (45.41)	3.46 ± 0.70		
**Research status**			56.823	< 0.001
Very good	27 (2.92)	3.91 ± 0.81		
Good	89 (9.62)	3.76 ± 0.65		
Average	627 (67.78)	3.25 ± 0.49		
Not good	117 (12.65)	2.94 ± 0.49		
Very bad	65 (7.03)	2.76 ± 0.61		

### 3.4. Analysis of factors influencing the satisfaction of research capacity development of Master of Public Health

The purpose of our analysis of variance for this part of the values was to identify statistically significant influences for the next part of the linear regression analysis. The results of univariate analysis showed that the length of practice in the unit, subsidy treatment, importance of scientific research ability training, development tendency, research system, incentive system, and current status of scientific research were statistically significant (*P* < 0.05), and gender, academic year, age, and whether or not it was a medical degree were not statistically significant (*P* < 0.05).

Multifactorial logistic regression analysis was performed with satisfaction with the development of research capacity of Master of Public Health students as the dependent variable, and indicators with statistically significant differences in the current status of research as the independent variables. The results showed that the length of practice in the unit, subsidized treatment, importance of research capacity development, development tendency, and current status of research were the factors influencing the satisfaction of Master of Public Health students with research capacity development (*P* < 0.05). See Table 4.

**Table 4:** Logistic regression analysis of factors influencing the satisfaction of Public Health Master's research ability training.

**Independent variable**	***b***	***SE***	**Waldχ^2^**	***P***	***OR***	**95% CI**
**Duration of practice in the practice unit (months, reference group: 3 months and below)**						
3–6 months	−1.388	2.190	0.402	0.026	0.250	(0.003, 18.254)
6 months and above	−2.631	2.208	1.420	0.033	0.072	(0.001, 5.455)
**Subsidized treatment (Yuan/month, reference group: 1,000 and below)**						
1,000–2,000	0.337	0.304	1.231	0.267	1.401	(0.772, 2.5421)
2,000–3,000	0.759	0.290	6.839	0.009	2.137	(1.210, 3.775)
3,000 and above	0.076	0.312	0.060	0.807	1.079	(0.586, 1.988)
**Importance of Master of Public Health students research capacity development (reference group: very important)**						
Important	−1.908	0.981	12.783	0.029	0.148	(0.02, 0.22)
General	−1.059	0.939	23.273	0.003	0.094	(0.06, 0.18)
Not important	−1.361	0.922	2.181	0.024	0.066	(0.04, 0.14)
Very unimportant	−1.079	0.951	0.007	0.033	0.023	(0.01, 0.04)
**Developmental tendencies (reference group: mainly practical skills)**						
Research ability as the main focus	3.077	0.403	58.443	< 0.001	21.695	(9.857, 47.749)
Research and practice are both important	0.867	0.408	4.526	0.033	2.380	(1.071, 5.292)
**Research status (reference group: very good)**						
Good	0.080	1.169	0.005	0.945	1.083	(0.110, 10.702)
Average	−1.416	0.607	5.451	0.020	0.243	(0.074, 0.797)
Not good	−1.587	0.613	6.709	0.010	0.205	(0.062, 0.680)
Very bad	−1.777	0.715	6.177	0.013	0.169	(0.042, 0.687)

## 4. Discussion

Unlike undergraduate generalized discipline education, the cultivation of postgraduate education is more important for the cultivation of innovative thinking and practical professional ability, which requires paying attention to individualized teaching and cultivation under the premise of taking into account the commonality (4). The fundamental difference between Master of Public Health and general public health-related undergraduates lies in their postgraduate status and matching scientific research ability. Cultivation of scientific research ability is the key to whether the Master of Public Health can become a high-level talent with innovative ability in the process of practice (5).

### 4.1. Satisfaction of Master of Public Health students with the training of scientific research ability

The overall mean score of the satisfaction of Master of Public Health students on research ability training is (3.29 ± 0.87), which reaches the score of basic satisfaction, but there is still an obvious gap with high satisfaction, and there are still some items that have more room for improvement. The area with the lowest satisfaction are the subsidized treatment, which may be due to the academic pressure and family economic pressure on the one hand, and the Master of Public Health students can only barely make ends meet or cannot make ends meet; On the other hand, it may be due to the peer pressure and the reason of the long academic training cycle.

The area with the highest satisfaction is the effect of faculty research guidance, with a satisfaction rate of 59.46%. Chen (6) conducted a questionnaire survey on 1,821 current graduate students, in which the satisfaction rate of supervised research was 83.55%. It was significantly higher than the present study, probably due to the difference in the study groups, which only included current master's degree students in public health, not doctoral students and academic degree students. In the study of Zou et al. (5, 7, 8), who conducted a survey on the satisfaction of scientific research capacity development in eight universities, the satisfaction with mentor's scientific research guidance, was consistent with the findings of this study.

### 4.2. Factors influencing the satisfaction of research competence development of Master of Public Health

The results of the study showed that, the positive correlation between the length of practice hours of Master of Public Health in practice units and their satisfaction with research competence can be attributed to the specificity of Master of Public Health competence development. In the practice unit, it may be more focused on problem-solving skills and scientific competence to accomplish research tasks rather than favoring the development of scientific competence. This is in line with the training purpose of the Master of Public Health, i.e., the requirement to train comprehensive public health personnel for practice and application (9).

Master of Public Health students who regarded the development of research ability as important had better satisfaction with their own research ability. Master of Public Health students who value the training of research ability are more likely to dedicate their time and energy to risorous research agendas. They will pay enough attention to and allocate their energy to the task of research, and consciously demonstrating the attitude for research and study, which is the internal driving force and an important prerequisite for the completion of the training of research ability (10). The results of the survey showed that among the students who considered research ability training important and very important, 359 (50.21%) wanted to obtain more research resources, and 399 (55.34%) thought that there was less training and hoped that there would be more research ability training.

Satisfaction with the development of research competence was significantly higher in the Master of Public Health students with a developmental tendency favoring research competence as the main focus than in the Master of Public Health students with a tendency favoring practical competence as the main focus. It may be because these Master of Public Health students themselves are more interested in the development of research ability and focus on the improvement of personal research ability. Master of Public Health students with better research pedigree also have significantly higher satisfaction with research ability development than those with insufficient research capabilities, which on the one hand may have benefited from the results of research ability training, coupled with a correct learning attitude and unremitting efforts, and therefore have good research results, consistent with the results of the study by Zhou et al. (11).

### 4.3. Suggestions to improve the satisfaction of research training for Master of Public Health

First of all, on the basis of the current training of scientific research ability of Master of Public Health, medical schools should increase the subsidy system adapted to the practice of Master of Public Health students (12), distribute according to labor, subsidize according to the value of the contribution of the actual scientific research project, and at the same time, can introduce more medicine-related social resources to carry out paid cooperation. While cultivating the scientific research ability of students, the construction of the system of subsidized treatment of students is guaranteed.

Secondly, medical schools should strengthen the construction of public health tutor team and improve the frequency and quality of scientific research exchanges between teachers and students. It has been proved that the mentor team is a key factor affecting the quality of Public Health Master's degree students' own training (13). Therefore, it is necessary to give full play to the important role of the mentor in the cultivation of scientific research ability of Master of Public Health. The communication between mentors and students, their own scientific research level, teacher ethics, and the way of guiding students are of great significance to the cultivation of graduate students' scientific research ability. Research communication between supervisors and Master of Public Health students also plays a pivotal role. It has been shown that there is a significant difference in the time to write a thesis between students with different frequency of communication with their supervisors (14).

In addition, colleges and universities should improve the existing research incentives and policies, adopt multiple forms and types of research incentives to promote the transformation of scientific research results, and promote the enthusiasm of Master of Public Health students in the cultivation of scientific research ability and scientific research output. Finally, colleges and universities should pay attention to the development of scientific research courses and scientific research training, and at the same time, based on the needs of social public health in the training courses, for the characteristics of the specialty of public health, to cultivate a group of high-quality scientific research talents in public health, so that the cultivation system is adapted to the development of the times.

## Data availability statement

The raw data supporting the conclusions of this article will be made available by the authors, without undue reservation.

## Ethics statement

The manuscript presents research on animals that do not require ethical approval for their study. Written informed consent was obtained from the individual(s) for the publication of any potentially identifiable images or data included in this article.

## Author contributions

BH: Formal analysis, Funding acquisition, Writing—original draft, Writing—review and editing. HZ: Data curation, Writing—original draft. YW: Writing—original draft, Writing—review and editing. LZ: Data curation, Methodology, Writing—original draft. ML: Formal analysis, Writing–original draft.
